# Deep RNA sequencing reveals the dynamic regulation of miRNA, lncRNAs, and mRNAs in osteosarcoma tumorigenesis and pulmonary metastasis

**DOI:** 10.1038/s41419-018-0813-5

**Published:** 2018-07-10

**Authors:** Lin Xie, Zhihong Yao, Ya Zhang, Dongqi Li, Fengdi Hu, Yedan Liao, Ling Zhou, Yonghong Zhou, Zeyong Huang, Zewei He, Lei Han, Yihao Yang, Zuozhang Yang

**Affiliations:** 1 Bone and Soft Tissue Tumors Research Center of Yunnan Province, Department of Orthopaedics, The Third Affiliated Hospital of Kunming Medical University (Tumor Hospital of Yunnan Province), Kunming, 650118 Yunnan China; 2 Department of Medical Oncology, The Third Affiliated Hospital of Kunming Medical University (Tumor Hospital of Yunnan Province), Kunming, 650118 Yunnan China; 3 Medical School, Kunming University of Science and Technology, Kunming, 650504 Yunnan China

## Abstract

Osteosarcoma (OS) is the most common pediatric malignant bone tumor, and occurrence of pulmonary metastasis generally causes a rapid and fatal outcome. Here we aimed to provide clues for exploring the mechanism of tumorigenesis and pulmonary metastasis for OS by comprehensive analysis of microRNA (miRNA), long non-coding RNA (lncRNA), and mRNA expression in primary OS and OS pulmonary metastasis. In this study, deep sequencing with samples from primary OS (*n* = 3), pulmonary metastatic OS (*n* = 3), and normal controls (*n* = 3) was conducted and differentially expressed miRNAs (DEmiRNAs), lncRNAs (DElncRNAs), and mRNAs (DEmRNAs) between primary OS and normal controls as well as pulmonary metastatic and primary OS were identified. A total of 65 DEmiRNAs, 233 DElncRNAs, and 1405 DEmRNAs were obtained between primary OS and normal controls; 48 DEmiRNAs, 50 DElncRNAs, and 307 DEmRNAs were obtained between pulmonary metastatic and primary OS. Then, the target DEmRNAs and DElncRNAs regulated by the same DEmiRNAs were searched and the OS tumorigenesis-related and OS pulmonary metastasis-related competing endogenous RNA (ceRNA) networks were constructed, respectively. Based on these ceRNA networks and Venn diagram analysis, we obtained 3 DEmiRNAs, 15 DElncRNAs, and 100 DEmRNAs, and eight target pairs including miR-223-5p/(CLSTN2, AC009951.1, LINC01705, AC090673.1), miR-378b/(ALX4, IGSF3, SULF1), and miR-323b-3p/TGFBR3 were involved in both tumorigenesis and pulmonary metastasis of OS. The TGF-β superfamily co-receptor TGFBR3, which is regulated by miR-323b-3p, acts as a tumor suppressor in OS tumorigenesis and acts as a tumor promoter in pulmonary metastatic OS via activation of the epithelial–mesenchymal transition (EMT) program.

In conclusion, the OS transcriptome (miRNA, lncRNA, and mRNA) is dynamically regulated. These analyses might provide new clues to uncover the molecular mechanisms and signaling networks that contribute to OS progression, toward patient-tailored and novel-targeted treatments.

## Introduction

Osteosarcoma (OS) is one of the main primary malignant bone tumor subtypes which mostly occurs in adolescents at sites of rapid bone growth^1^. Although intensive efforts to improve both chemotherapeutics and surgical management have been made, the high local aggressiveness and rapid metastasizing potential to lung results in poor survival for patients with OS. Therefore, the ultimate treatment depends on primary OS control and the removal of small metastases. OS is a pathology that affects bone remodeling, involving alterations in both osteoblast and osteoclast functions. However, the mechanisms underlying its initiation and progression remain unclear.

Non-coding RNAs (ncRNAs) have no ability of coding proteins while they can act as functional RNAs. Based on the transcript size, ncRNAs are grouped into small ncRNAs (<200 bp) and long ncRNAs (>200 bp, up to 100 kb). MicroRNAs (miRNAs), a class of small ncRNAs (≈22 nt), are crucial to the regulation of gene expression through partial base-pairing with target mRNAs. They have multiple roles in various biological processes that affect basic cellular functions, including cell proliferation, differentiation, death, and tumorigenesis^2^. Unlike miRNAs, long non-coding RNA (lncRNAs) play critical and complicated roles in the regulation of various biological processes, including chromatin modification, transcription, and post-transcriptional processing^3^. Currently, growing evidences indicated that there are interactions between lncRNAs and miRNAs, the downstream target genes of which have been closely related to tumor pathogenesis.

RNA sequencing (RNA-seq) has been used widely to study specific gene expression patterns at different developmental stages. In this study, we obtained the miRNA, mRNA, and lncRNA expression data from normal controls, primary OS, and pulmonary metastatic OS based on RNA-seq, and we constructed the competing endogenous (ceRNA) network to elaborate the interactions and potential crosstalk between the differentially expressed hub lncRNAs (DElncRNAs), miRNAs (DEmiRNAs), and mRNAs (DEmRNAs).

## Materials and methods

### Sample preparation

In the present study, we recruited patients with OS from the Third Affiliated Hospital of Kunming Medical University. Detailed information of patients is displayed in Table 1. The fresh tumor tissues were obtained from the primary lesion of three patients with primary OS (P1–P3) and three patients with pulmonary metastatic OS (M1–M3) after surgical resection. The control non-cancerous tissues were obtained from distal tumor location of three patients with primary OS (N1–N3). Tissue samples were frozen in liquid nitrogen and stored at −80 °C before RNA isolation. The present study complied with Declaration of Helsinki and was approved by the Institutional Review Boards of the Third Affiliated Hospital of Kunming Medical University. Moreover, all subjects provided written informed consent.

**Table 1:** Patient characteristics

Index	N1	N2	N3	P1	P2	P3	M1	M2	M3
Age	15	22	19	22	16	30	13	33	34
Gender	Female	Male	Male	Male	Male	Female	Male	Female	Female
Grade	G2	G2	G2	G2	G2	G2	G2	G2	G2
TM stage	T2M0	T2M0	T2M0	T2M0	T2M0	T2M0	T2M1	T2M1	T2M1
Site of metastasis	–	–	–	–	–	–	Lung	Lung	Lung

### RNA extraction and quality monitoring

All the surgical specimens were subjected to RNA extraction using the Trizol reagent (Invitrogen, Carlsbad, CA, USA) according to the manufacturer’s protocol. The RNA quality was evaluated with the NanoDrop2000 Spectrophotometer (Thermo Fisher Scientific, Wilmington, DE, USA) and Agilent 2100 Bioanalyzer (Agilent Technologies). Purified RNA was stored at −80 °C until required.

### Small RNA library construction, sequencing, and data processing

Following extraction and purification, about 1 μg total RNA per sample was used to construct the small RNA (sRNA) library using TrueSeq small RNA library prep kit (Illumina San Diego CA, USA) according to the manufacturer’s instruction. Adapters were ligated to the 3′ end of the RNA, followed by the ligation of the 5′ adapter. Subsequently, the RNA was reverse transcribed to create single-stranded cDNA, followed by single-end sequencing (50 base pairs in length) on an Illumina on the HiSeq4000 sequencer (Illumina, San Diego, CA, USA).

Raw data (raw reads) were processed with an in-house pipeline consisting of adapter trimming, read alignment and read counting. The trimmed reads, also known as clean reads, were mapped to the human reference genome GRCh38 using the popular alignment tool Bowtie^4^. Then, the modified software miRDeep2 (https://www.mdc-berlin.de/8551903/en/) was used to compute miRNA read counts^5^. Mature miRNA and miRNA precursors were downloaded from miRBase. Moreover, the differentially expressed miRNAs (DEmiRNAs) between samples were identified using DEGseq package in R. The *p*-value <0.01 and |log2 (Fold_change)| > 2 were used as the cut-off criteria.

### lncRNA + mRNA sequencing and data processing

A total of 3 μg RNA per sample was used for the RNA sample preparations. After removing the ribosomal RNA, the rRNA-depleted RNA was fragmented and the cDNA library was constructed using the Truseq RNA sample Prep Kit (Illumina, Inc., San Diego, CA, USA). The libraries were sequenced on an Illumina Hiseq 2500 platform (Illumina Inc., San Diego, CA, USA) according to the manufacturer’s instructions and 125 bp paired-end reads were generated.

Raw reads of fastq format were then processed through in-house perl scripts. After triming the raw reads, we obtained the clean reads, which were mapped to the human reference genome Ensembl V84 using Tophat. The mapped reads were quantified with cuffquant, and the differentially expressed mRNAs (DEmRNAs) and differentially expressed lncRNAs (DElncRNAs) between samples were identified using Cuffdiff program from the Cufflinks package. The p-value < 0.01 and |log2 (Fold_change)| > 2 were used as the cut-off criteria.

### Function enrichment analysis

We performed Gene Ontology (GO) enrichment and Kyoto Encyclopedia of Genes and Genomes (KEGG) pathway analyses on these DEmRNAs and predicted target genes of DEmiRNAs and DElncRNAs. GO term and KEGG pathway analyses of coding genes were performed using GeneCodis3 bioinformatics resources^6^. Both GO terms and KEGG pathways with corrected. *p*-Values <0.05 were considered to be significantly enriched.

### Schema for integrative analysis of DEmiRNAs, DEncRNA, and DEmRNA

Systematic bioinformatic analysis was developed based on possible functional relationships between DEmiRNAs, DEncRNA, and DEmRNA. Firstly, by scanning for conserved miRNA target sites with RNA22, miRanda, miRDB, miRWalk, PICTAR2, and Targetscan, we predicted the target genes and target lncRNAs for the DEmiRNAs. Secondly, we searched coding genes within the 100-kb upstream and downstream regions of each DElncRNAs and found the cis-acting genes. According to the functional relationships between these molecules, the miRNA-target gene regulatory network, miRNA–lncRNA target regulatory network, lncRNA–mRNA co-expression network were established, respectively. Next, we constructed the ceRNA network.

### Quantitative real-time polymerase chain reaction (qRT-PCR)

To validate the expression levels of the selected lncRNAs by qRT-PCR, RNA samples from the additional 36 individuals with primary OS and 33 individuals with pulmonary metastatic OS were collected. To validate the expression levels of selected genes and miRNAs by qRT-PCR, RNA samples from the additional 30 individuals with primary OS and 27 individuals with pulmonary metastatic OS were collected. Total RNA was isolated by using the Trizol reagent (Invitrogen, USA) according to manufacturer’s protocol. The mRNA template was reversely transcribed into cDNA using reverse transcriptase Kit (TaKaRa, Dalian, China). The miRNA reverse transcription was performed using miRcute miRNA First-strand cDNA Synthesis kits (TIANGEN, China). Forward and reverse primers were designed and qRT-PCR was carried out on BIO-RAD IQ5 RT-PCR Detection System (Bio-Rad Laboratories Inc., Germany). The expression levels of the selected lncRNAs and miRNAs were normalized against the snU6. The expression levels of the selected genes were normalized against GAPDH.

### Cross-validation

The miRNA expression data of GSE65071 was downloaded from GEO database (https://www.ncbi.nlm.nih.gov/geo/), including 20 plasma samples from OS cases and 15 plasma samples from controls plasma. The DEmiRNAs were validated between comparison of case group and control samples. Moreover, the mRNA data of GSE14359 was also downloaded from GEO database (https://www.ncbi.nlm.nih.gov/geo/), including 10 conventional OS tissues and eight OS lung metastasis tissues. The DEmRNAs were validated between comparison of OS lung metastasis group and OS group.

## Results

### Sequencing and mapping of the OS transcriptome

**Fig. 1:**
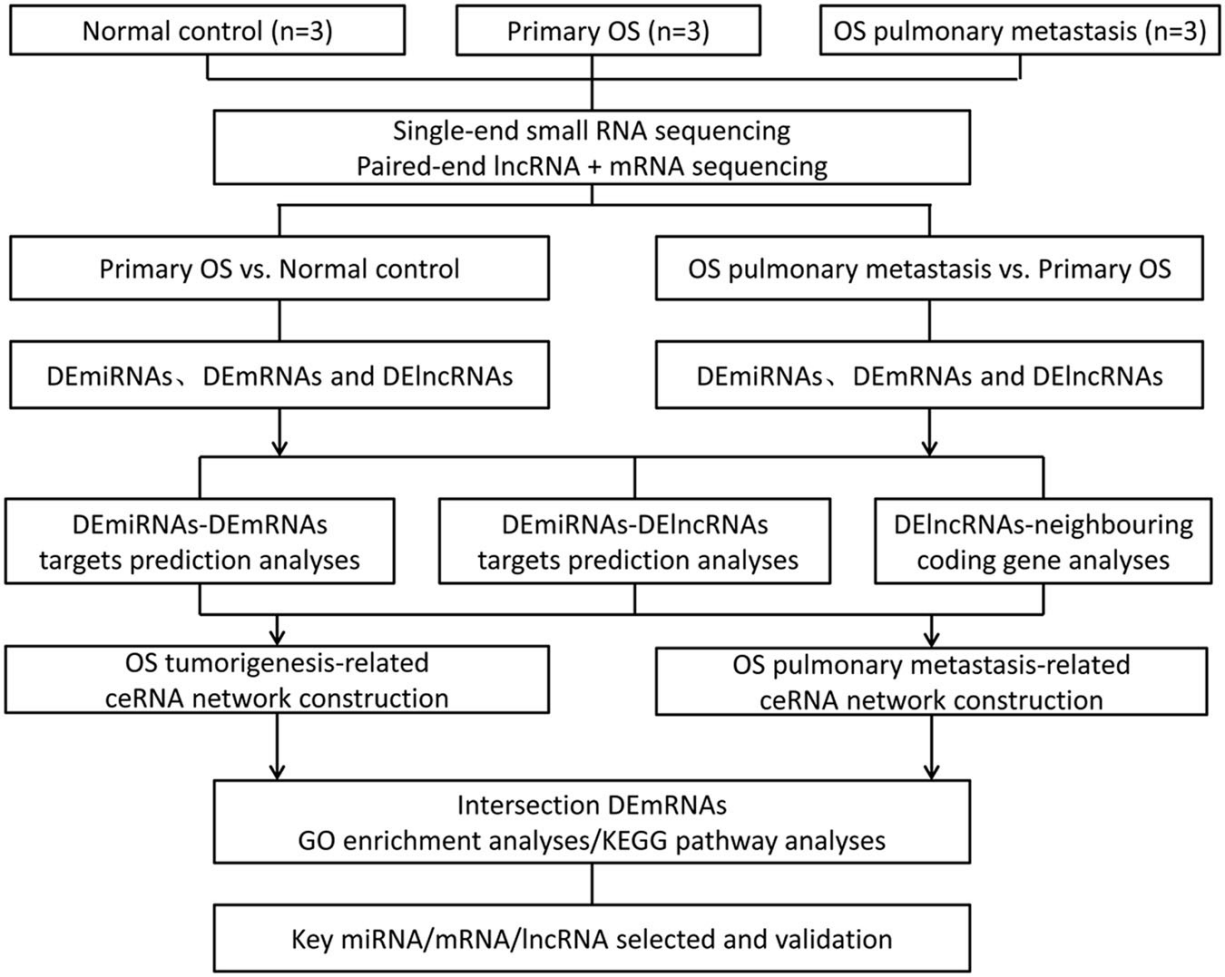
Comprehensive analyses workflow of miRNAs, mRNAs, and lncRNAs in human primary OS, pulmonary metastatic OS, and normal controls

We sequenced the cDNA and sRNA libraries of nine tissue samples from three primary OS patients, three pulmonary metastatic OS patients, and three controls. Counts of clean reads and mapped ratio of sequencing results were displayed in Supplemental Table S1. The overall workflow is shown in Fig. 1.

### Deep RNA-seq revealed distinct expression signatures of coding and ncRNAs in OS progression

**Fig. 2:**
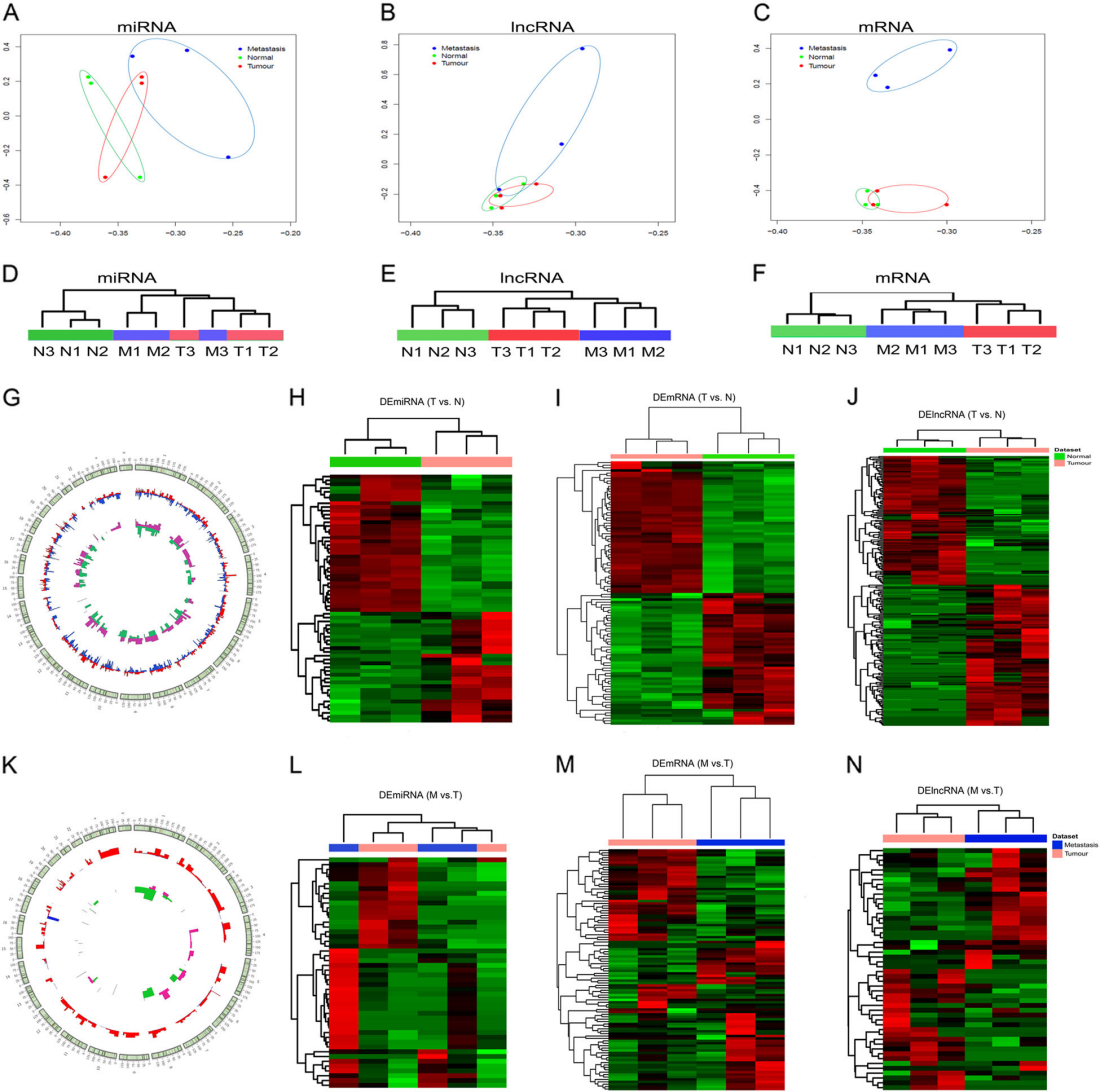
RNA-seq reveals distinct expression pattern of miRNAs, lncRNAs, and mRNAs in human primary OS, pulmonary metastatic OS, and normal controls. **a**–**c** Principal component analyses of miRNA, lncRNA, and mRNA expression profiles. **d**–**f** Unsupervised hierarchical clustering of the expression profiles of DEmiRNAs, DElncRNAs, and DEmRNAs. **g** Circus plot representing the distribution of DElncRNAs and DEmRNAs in primary OS vs. normal controls on chromosomes. **h**–**j** Unsupervised clustering analysis showing expression profiles of DEmiRNAs, DEmRNAs, and DElncRNAs in primary OS vs. normal controls. **k** Circus plot representing the distribution of DElncRNAs and DEmRNAs in pulmonary metastatic OS vs. primary OS on chromosomes. **l**–**n** Unsupervised clustering analysis showing expression profiles of DEmiRNAs, DEmRNAs, and DElncRNAs in pulmonary metastatic OS vs. primary OS

Principal component analyses (PCA) revealed that miRNA, lncRNA, and mRNA expression profiles distinguish primary OS, pulmonary metastatic OS from the controls (Fig. 2a–c). Using the criterion of *p* < 0.01 and |log2(fold change)| > 2, we detected 65 DEmiRNAs, 233 DElncRNAs, and 1405 DEmRNAs in primary OS compared with the normal controls. Top ten miRNAs, lncRNAs, and mRNAs exhibiting significant up- and downregulation are listed in Table 2. Totally, we detected 48 DEmiRNAs, 50 DElncRNAs, and 307 DEmRNAs in pulmonary metastatic OS compared with primary OS. Top ten miRNAs, lncRNAs, and mRNAs exhibiting significant up- and downregulation are listed in Table 2. Unsupervised hierarchical clustering of the DEmiRNAs, DElncRNAs, and DEmRNAs (Fig. 2d–f) revealed a distinct expression signature of all three RNA species in primary OS and OS pulmonary metastasis, compared to the control samples.

**Table 2:** Top ten up- and downregulated miRNAs, lncRNAs, and mRNAs in primary OS and OS pulmonary metastasis

Primary OS									
Differentially expressed miRNAs			Differentially expressed lncRNAs				Differentially expressed mRNAs		
Gene symbol	*p*-Value	log2FC	Ensembl Gene ID	Gene symbol	*p*-Value	log2FC	Gene symbol	*p*-Value	log2FC
*Upregulation*			*Upregulation*				*Upregulation*		
miR-2682-5p	0.002005	8.66	ENSG00000222032	AC112721.2	5.00E−05	7.76	SPP1	0.00105	10.93
miR-4435	0.009722	7.08	ENSG00000271857	AL096865.1	5.00E−05	6.82	ACP5	5.00E−05	8.93
miR-181b-3p	6.99E−05	7.05	ENSG00000197301	LOC100129940	5.00E−05	6.74	PCLAF	0.00065	6.91
miR-138-1-3p	0.006732	6.59	ENSG00000263707	AC005277.1	5.00E−05	6.23	COL1A1	5.00E−05	6.89
miR-4741	0.002851	6.39	ENSG00000225107	AC092484.1	5.00E−05	6.11	PIMREG	1.00E−04	6.81
miR-4421	0.005265	6.29	ENSG00000231290	APCDD1L-DT	5.00E−05	5.86	INSC	3.00E−04	6.78
miR-940	0.000135	5.76	ENSG00000257045	AC016746.1	5.00E−05	5.83	DGKI	0.00165	6.71
miR-224-5p	0.002128	5.66	ENSG00000275097	AC024940.6	5.00E−05	5.77	TENM4	5.00E−05	6.57
miR-222-3p	6.87E−05	5.58	ENSG00000232679	LNC01705	5.00E−05	5.72	PTPN22	1.00E−04	6.52
miR-203a-3p	0.009058	5.58	ENSG00000242590	AL645608.6	5.00E−05	5.68	DMP1	5.00E−05	6.33
*Downregulation*			*Downregulation*				*Downregulation*		
miR-1-3p	1.80E−38	−10.5	ENSG00000224609	HSD52	5.00E−05	−9.35	MYBPC1	1.00E−04	−13.73
miR-133a-3p	4.54E−43	−10.19	ENSG00000272446	AL158850.2	1.00E−04	−8.35	MYH7	5.00E−05	−13.06
miR-208b-3p	2.10E−14	−9.9	ENSG00000265142	MIR133A1HG	5.00E−05	−8.08	MYH1	5.00E−05	−12.91
miR-206	1.10E−20	−9.5	ENSG00000258283	AC011603.3	5.00E−05	−7.92	MYH2	0.002	−12.88
miR-133a-5p	3.16E−20	−9.46	ENSG00000256609	AC084880.3	5.00E−05	−7.54	TNNT1	5.00E−05	−11.92
miR-499a-5p	2.85E−16	−9	ENSG00000265751	AC015878.1	5.00E−05	−7.41	TNNI2	5.00E−05	−11.28
miR-95-5p	2.10E−07	−8.8	ENSG00000229444	LOC101929592	5.00E−05	−7.32	ANKRD2	0.0027	−10.81
miR-128-2-5p	2.03E−05	−7.55	ENSG00000235050	MLIP-AS1	5.00E−05	−7.1	TNNC1	0.00095	−10.53
miR-6505-5p	6.41E−08	−7.05	ENSG00000257514	AC117505.1	5.00E−05	−6.76	ATP2A1	5.00E−05	−10.22
miR-520b	0.008803	−5.3	ENSG00000236208	C10ORF71-AS1	5.00E−05	−6.63	CMYA5	5.00E−05	−10.21
OS pulmonary metastasis									
Differentially expressed miRNAs			Differentially expressed lncRNAs				Differentially expressed mRNAs		
Gene symbol	*p*-Value	log2FC	Ensembl Gene ID		*p*-Value	log2FC	Gene symbol	*p*-Value	log2FC
*Upregulation*			*Upregulation*				*Upregulation*		
hsa-miR-3653-3p	0.00139	8.36	ENSG00000238121	LINC00426	1.55E−03	5.429108	C4B	5.00E−05	6.709397
hsa-miR-4783-3p	0.003224	7.71	ENSG00000249219	lnc-DRD5-1	5.00E−05	4.753273	LIPE	5.00E−05	5.931086
hsa-miR-3144-3p	0.005005	7.1	ENSG00000243193	LNC-COG5-2	6.65E−03	4.627595	GAGE12C	5.00E−05	5.825597
hsa-miR-1269a	0.000269	7.01	ENSG00000279221	AC068254.2	5.00E−05	3.664665	CKMT1B	1.00E−04	5.45318
hsa-miR-1262	0.001956	6.08	ENSG00000260254	AP000997.2	1.30E−03	3.654228	DGAT2	0.0093	5.247579
hsa-miR-4725-3p	0.001195	5.8	ENSG00000267549	Lnc-ZNF583-5	1.10E−03	3.467332	KCNK3	5.00E−05	5.206908
hsa-miR-4651	0.00269	5.66	ENSG00000228791	THRB-AS1	2.40E−03	3.304355	SOD3	5.00E−05	5.130038
hsa-miR-4443	0.005788	5.51	ENSG00000229776	C4B-AS1	4.35E−03	3.274502	ITGA7	0.00085	5.124185
hsa-miR-760	0.003071	4.96	ENSG00000225138	SLC9A3-AS1	5.00E−05	3.243303	RIMS2	5.00E−05	5.016506
hsa-miR-4507	0.006545	4.89	ENSG00000279692	AC110285.7	3.20E−03	3.177157	KRT8	5.00E−04	4.979941
*Downregulation*			*Downregulation*				*Downregulation*		
hsa-miR-539-3p	0.000284	−9.69	ENSG00000274370	lnc-METRNL-8	5.00E−05	−6.05459	SPATA22	0.00525	−159.879
hsa-miR-612	0.006778	−9.35	ENSG00000275327	lnc-MGMT-12	1.00E−04	−5.84527	CTSK	5.00E−05	−7.01673
hsa-miR-1197	0.005934	−9.19	ENSG00000232679	lnc-DUSP10-4	5.00E−05	−5.71626	NDNF	5.00E−05	−6.31953
hsa-miR-655-3p	0.00361	−8.94	ENSG00000259196	HMBOX1-IT1	1.00E−04	−4.91397	CHAD	0.00235	−5.52639
hsa-miR-889-3p	2.58E−05	−8.12	ENSG00000267886	lnc-ZNF730-6	1.55E−03	−4.7872	PRKN	0.0066	−5.26132
hsa-miR-381-3p	0.000726	−5.5	ENSG00000261040	WFDC21P	5.00E−05	−4.53685	FNDC1	5.00E−05	−5.19914
hsa-miR-1185-1-3p	0.000492	−5.39	ENSG00000254101	LINC02055	5.00E−05	−4.33215	FST	5.00E−05	−5.06677
hsa-miR-411-5p	0.000964	−5.3	ENSG00000233682	AL356417.2	1.45E−03	−4.15989	RUFY4	5.00E−05	−4.88628
hsa-miR-495-3p	0.000695	−5.19	ENSG00000281937	Z98044.1	3.00E−04	−4.00363	ENPP1	1.00E−04	−4.87775
hsa-miR-539-3p	0.002104	−5.13	ENSG00000197301	LOC100129940	1.15E−03	−3.995	DSP	2.00E−04	−4.75571

The distribution of DElncRNAs and DEmRNAs in primary OS compared with the normal controls is illustrated in Fig. 2g. The unsupervised clustering showed two robust clusters: one cluster encompassing all of primary OS and another cluster containing all of the controls. This indicated that tumors and controls might have different expression patterns (Fig. 2h–j). Moreover, the chromosomes distribution of DElncRNAs and DEmRNAs in pulmonary metastatic OS compared with primary OS is illustrated in Fig. 2k. Unsupervised hierarchical clustering of the expression profiles of mRNAs, miRNAs, and lncRNAs (Fig. 2l–n) revealed that lncRNA and mRNA expression profiles can largely distinguish the pulmonary metastatic OS and primary OS.

### OS tumorigenesis-related ncRNAs and pathways

Of the 65 DEmiRNAs, over 30 were reported to be associated with OS tumorigenesis, such as miR-1-3p^7^, miR-133a-3p^8^, miR-133a-5p^8^, miR-208b-3p^9^, miR-206^10^, miR-95-5p^11^, miR-128-2-5p^12^, miR-520b^13^, miR-520c-3p^14^, miR-378b^15^, miR-378h^15^, miR-378c^15^, miR-378e^15^, miR-378g^15^, miR-378a-5p^15^, miR-378i^15^, miR-30a-3p^16^, miR-30a-5p^16^, miR-422a^17^, miR-29b-3p^18^, miR-29a-3p^19^, miR-29c-5p^20^, miR-21-5p^21^, miR-155-5p^22^, miR-449a^23^, miR-223-5p^24^, miR-130b-5p^25^, miR-181a-3p^26^, miR-92b-5p^27^, miR-31-5p^28^, let-7a-2-3p^29^, miR-224-5p^30^, miR-138-1-3p^31^, and miR-181b-3p^32^. Unlike the DEmiRNAs, we found that most of the 233 DElncRNAs were with unknown function. Specially, CDKN2B-AS1 (ENSG00000240498) was reported to be associated with OS^33^. Functional annotation of DEmRNAs showed that the most enriched biological processes were skeletal system development, ossification, positive regulation of cartilage development, intramembranous ossification, osteoclast differentiation, etc. Pathways in cancer, osteoclast differentiation, rheumatoid arthritis, Toll-like receptor signaling pathway, p53 signaling pathway, Wnt signaling pathway, Jak-STAT signaling pathway may be closely involved in OS tumorigenesis.

### OS tumorigenesis-related ceRNA network

mRNAs targeted by DEmiRNAs according to the miRNA–mRNA binding data from computational prediction and experimental validation databases were searched. Totally, we obtained 2448 possible miRNA–mRNA target pairs (Supplemental Fig. 1). Five significant miRNAs, miR-520c-3p (degree = 115), miR-30a-5p (degree = 111), miR-520b (degree = 111), miR-940 (degree = 101), and miR-548j-5p (degree = 95) had the most target genes. Significantly, there were 16 miRNA–mRNA pairs which may play crucial roles in OS tumorigenesis, such as miR-133a-3p and its target genes SERPINH1^34^, TPM4^35^, MRC2^36^, STK17B^37^, CD33^38^ and PIK3CG^39^, miR-378a-5p and RAD51^40^, miR-378b and target genes UHRF1^41^ and SRC^42^, miR-378c and PIK3CG^39^, UHRF1^41^ and SRC^42^, miR-378f and UHRF1^41^, miR-378g and NOTCH2^43^, miR-378h and UHRF1^41^, and miR-449a and SATB1^44^.

In the next step, we focused on whether these DEmiRNAs would target the DElncRNAs. Totally, miRNA–lncRNA target prediction analyses identified 909 miRNA–lncRNA target pairs (Supplemental Fig. 2). In the corresponding miRNA–lncRNA target regulatory network miR-130b-5p (degree = 59), miR-30a-3p (degree = 58), miR-206 (degree = 54), miR-181b-3p (degree = 51), and miR-29a-3p (degree = 49) had the most target lncRNAs. Further co-expression analyses indicated that most of the miRNA–lncRNA target pairs had sense relationships, and miRNA–lncRNA pairs were prone to be located on the same strands.

Previous studies have reported that lncRNAs may act in cis and affect the gene expression of their chromosomal neighborhood^45^, and most lncRNA transcripts can also be derived from divergent transcription^46^. We annotated the location relationship between each lncRNA and its cis target genes and obtained 125 lncRNAs and their neighboring genes pairs in total. Accordingly, the lncRNA–mRNA networks were constructed and visualized (Supplemental Fig. 3).

**Fig. 3:**
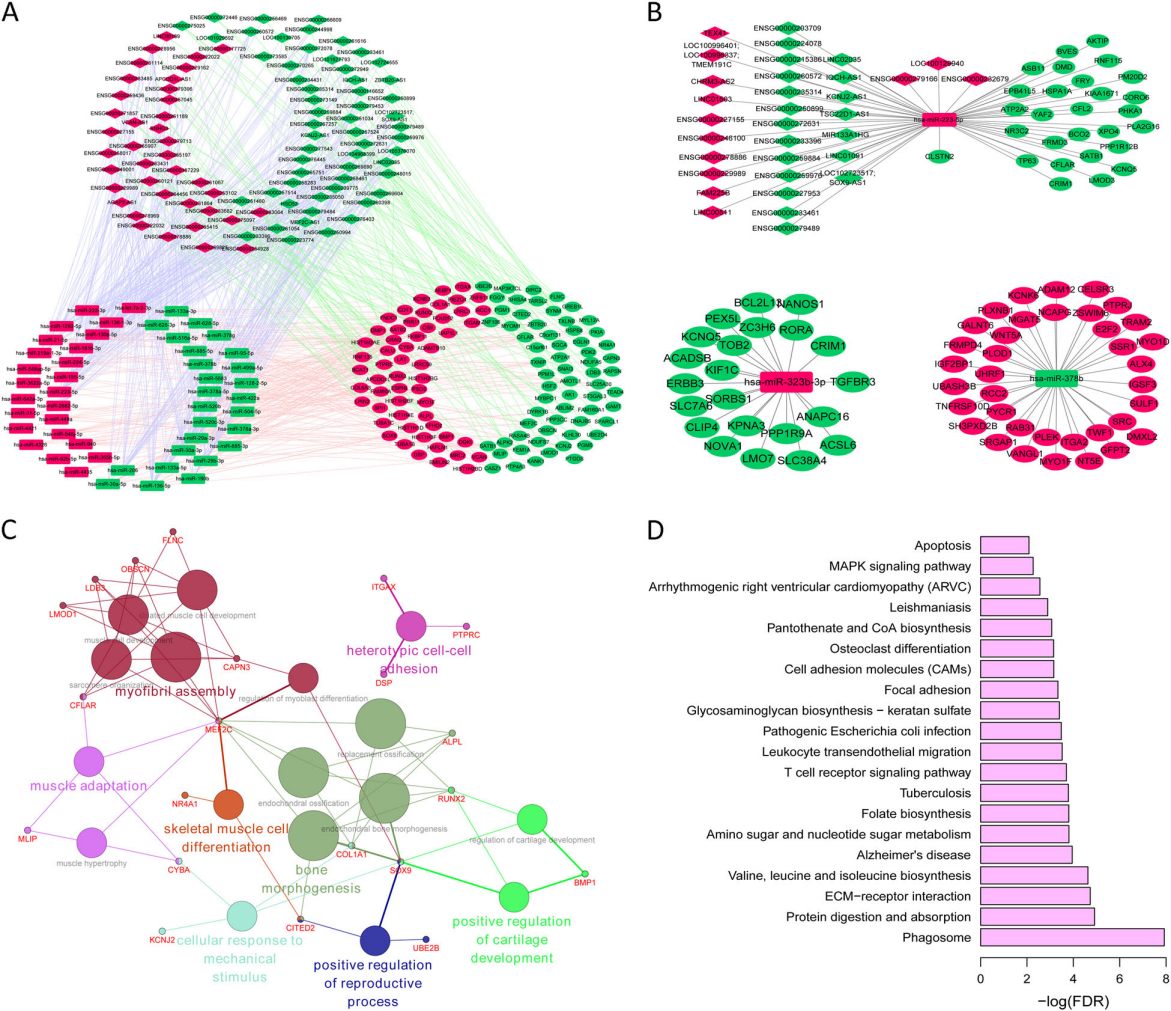
The OS tumorigenesis-related ceRNA network and their characteristics. **a** Global view of the ceRNA network. This network consists of 96 DElncRNAs, 50 DEmiRNAs, and 125 DEmRNAs. **b** Subnetworks of miR-223-5p, miR-378b, and miR-323b-3p. In Fig. 4a, b, rectangles, diamonds, and ellipses represented DEmiRNAs, DElncRNAs, and DEmRNAs between primary OS and normal controls, respectively. Red and green color represented upregulation and downregulation in primary OS compared to normal controls. **c** The functional enrichment map of GO terms. **d** Significantly enriched KEGG pathway of mRNAs in the ceRNA network

According to the target pairs of miRNA–mRNA, miRNA–lncRNA, and lncRNA-cis target gene, we constructed a ceRNA network (Fig. 3a). In particular, three miRNAs (miR-223-5p, miR-378b, and miR-323b-3p) were not only DEmiRNAs between primary OS and normal control but also DEmiRNAs between pulmonary metastasis OS and primary OS, which suggested that these three miRNAs might involve in both oncogenesis and metastasis of OS. Hence, subnetworks of miR-223-5p, miR-378b, and miR-323b-3p were shown in Fig. 3b. Functional annotation of mRNAs in the ceRNA network found that these mRNAs were mainly involved in seven GO terms (Fig. 3c), and KEGG pathways including Phagosome, ECM–receptor interaction, and two well-established cancer pathways, apoptosis^47^ and MAPK signaling pathway^48^ (Fig. 3d and Supplementary Table S2).

### OS pulmonary metastasis-related ncRNAs and pathways

Among the DEmiRNAs, 16 DEmiRNAs may be closely related to OS pulmonary metastasis, such as miR-539-3p^8^, miR-223-5p^24^, miR-381-3p^49^, miR-495-3p^50^, miR-144-3p^51^, miR-494-3p^52^, miR-433-3p^53^, miR-382-3p^54^, miR-329-3p^55^, miR-144-5p^51^, miR-134-5p^56^, miR-183-5p^57^, miR-20b-5p^58^, miR-101-3p^59^, miR-182-5p^60^, and miR-210-3p^61^. Most of the 50 DElncRNAs were with unknown function, in which only MEG3 (ENSG00000214548) was reported to be associated with the migration of OS^62,63^. Moreover, the functional enrichment of 307 DEmRNAs showed that most enriched biological processes were signal transduction, angiogenesis, type I interferon-mediated signaling pathway, cell adhesion, skeletal system development, and cell migration. Pathway analysis indicated that focal adhesion, ECM–receptor interaction, Amebiasis, complement and coagulation cascades, regulation of actin cytoskeleton, vascular smooth muscle contraction, *Staphylococcus aureus* infection, and rheumatoid arthritis may be closely involved in OS pulmonary metastasis.

### OS pulmonary metastasis-related ceRNA network

Totally, we obtained 318 possible miRNA–mRNA target pairs, and miRNA-target gene regulatory network is shown in Supplemental Fig. 4. We found that miR-612 (degree = 32), miR-182-5p (degree = 19), miR-20b-5p (degree = 17), miR-329-3p (degree = 15), and miR-495-3p (degree = 13) were closely related with these target DEmRNAs.

Next, the miRNA–lncRNA target prediction analyses identified 196 miRNA–lncRNA target pairs (Supplemental Fig. 5). In the corresponding miRNA–lncRNA target regulatory network, miR-182-5p (degree = 20), miR-20b-5p (degree = 16), miR-1262 (degree = 16), miR-134-5p (degree = 15), and miR-1197 (degree = 15) had the most target lncRNAs.

We further annotated the location relationship between each lncRNA and its cis target genes and obtained eight lncRNAs and their neighboring gene pairs in total, including C4B-AS1 (ENSG00000229776) and C4B, C4A-AS1 (ENSG00000233627) and C4B, AL356417.2 (ENSG00000233682) and FNDC1, AC020656.1 (ENSG00000257764) and LYZ, AL031058.1 (ENSG00000261189) and DSP, AC007686.3 (ENSG00000273729) and IRF2BPL, AL354950.2 (ENSG00000275327) and EBF3, AC122688.3 (ENSG00000279233) and AACS.

**Fig. 4:**
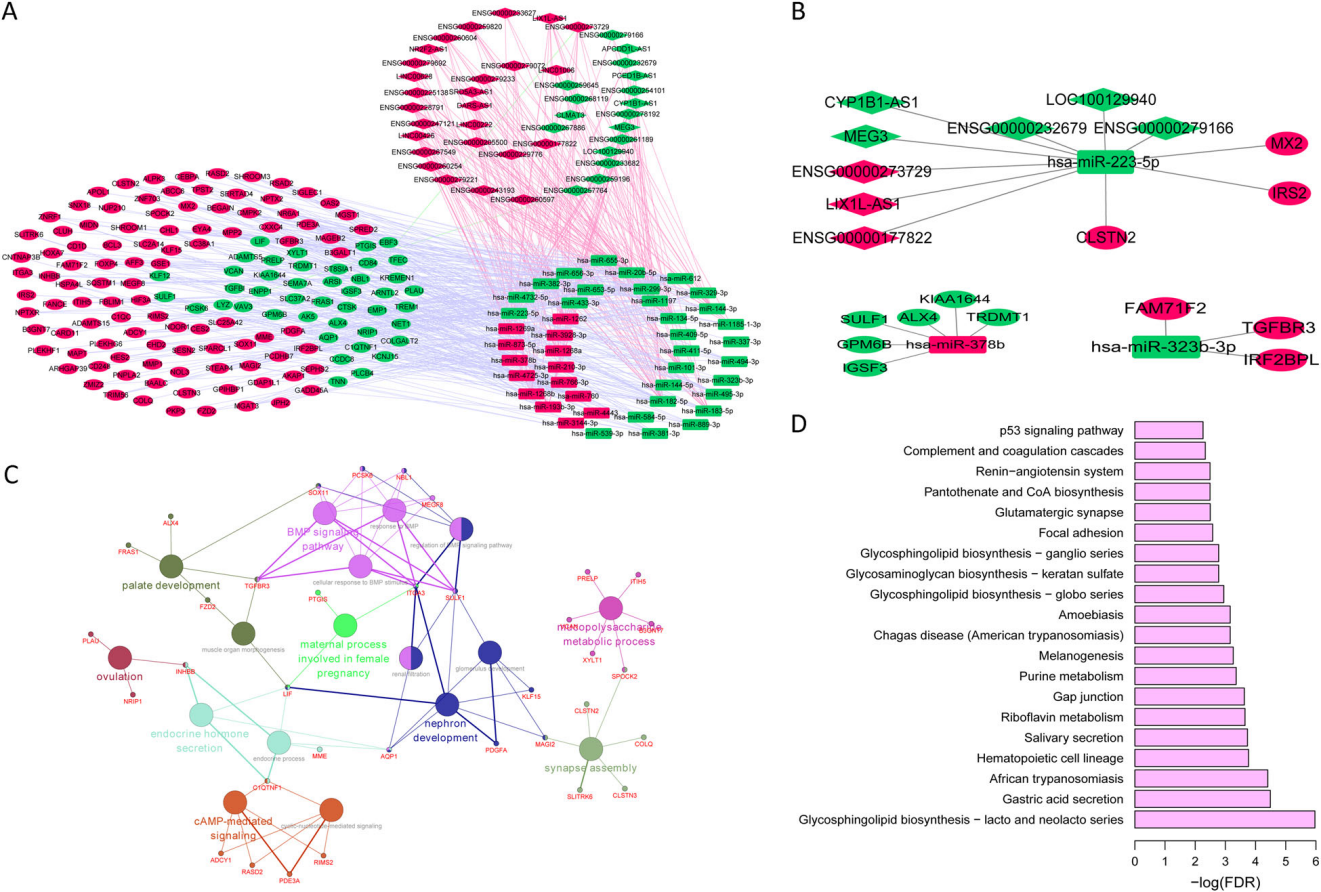
The OS pulmonary metastasis-related ceRNA network and their characteristics. **a** Global view of the ceRNA network. This network consists of 43 DElncRNAs, 43 DEmiRNAs, and 143 DEmRNAs. **b** Subnetworks of miR-223-5p, miR-378b, and miR-323b-3p. In Fig. 5a, b, rectangles, diamonds, and ellipses represented DEmiRNAs, DElncRNAs, and DEmRNAs between pulmonary metastasis and primary OS, respectively. Red and green color represented upregulation and downregulation in pulmonary metastasis OS compared to primary OS. **c** The functional enrichment map of GO terms. **d** Significantly enriched KEGG pathway of mRNAs in the ceRNA network

Totally, 43 lncRNAs, 43 miRNAs, and 143 mRNAs were involved in the ceRNA network (Fig. 4a). Since miR-223-5p, miR-378b, and miR-323b-3p were differentially expressed between primary OS and normal controls as well as pulmonary metastasis OS and primary OS. Subnetworks of miR-223-5p, miR-378b, and miR-323b-3p are shown in Fig. 4b. Functional annotation revealed that mRNAs in the ceRNA network were mainly involved in seven GO terms (Fig. 4c), and KEGG pathways including Hematopoietic cell lineage, Gap junction, Focal adhesion, Complement and coagulation cascades and a well-established cancer pathway, p53 signaling pathway^64^ (Fig. 4d and Supplementary Table S2).

### Several DEmiRNAs, DElncRNAs, and DEmRNAs play crucial roles in OS tumorigenesis and OS pulmonary metastasis

**Fig. 5:**
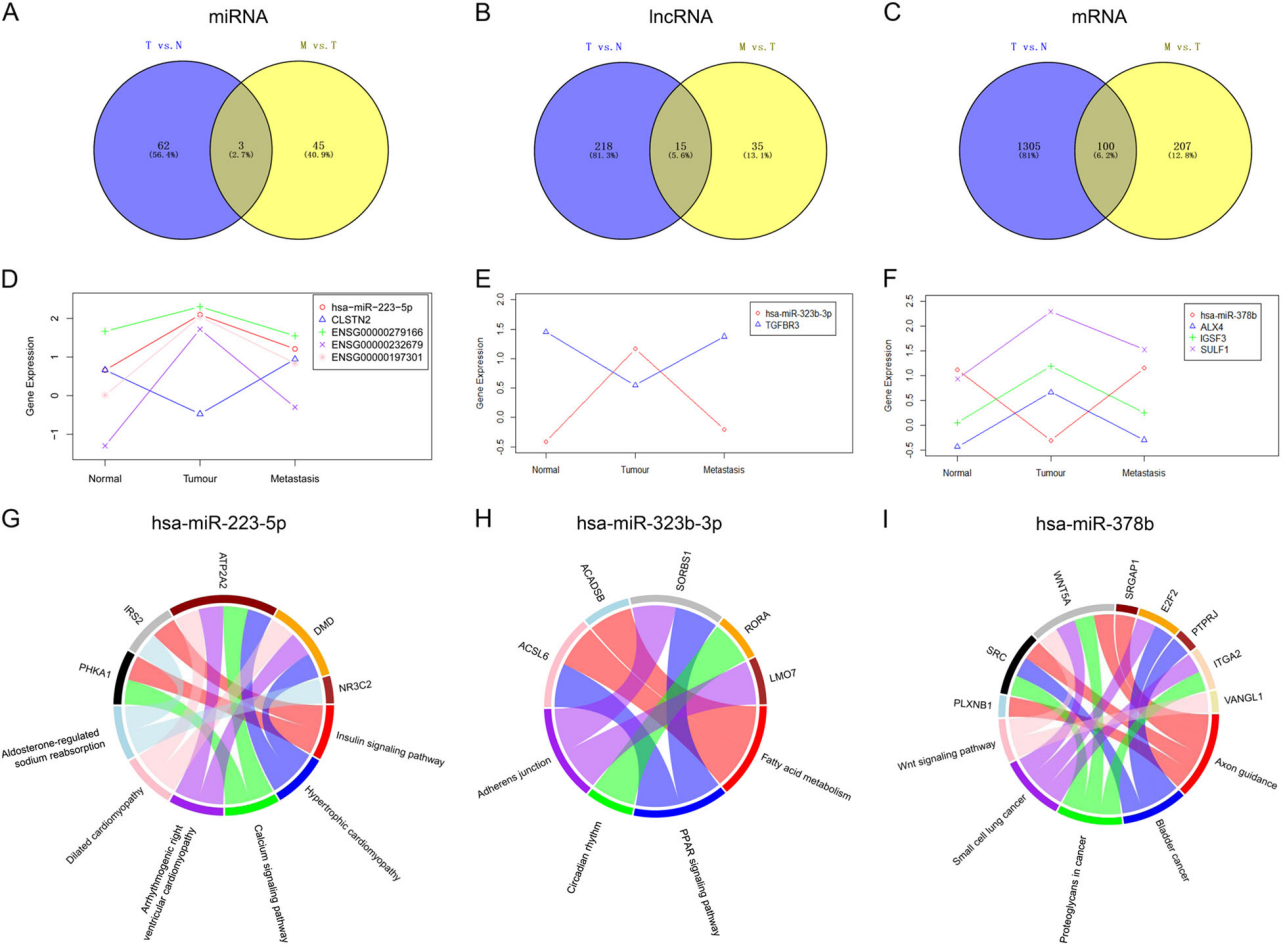
Validation of DElncRNA, DEmiRNA, and DEmRNA. The DElncRNAs were validated by qRT-PCR. Relative expression of DElncRNAs was determined by qRT-PCR of 36 primary OS samples and 33 pulmonary metastatic OS samples. DEmiRNA was validated using GSE65071 from GEO database (20 plasma from osteosarcoma cases and 15 controls plasma). DEmRNAs were validated by GSE14359 from GEO database (10 conventional osteosarcoma tissues and eight osteosarcoma lung metastasis tissues)

Venn diagram analysis of DEmiRNAs, DElncRNAs, and DEmRNAs was performed between primary OS vs. control and pulmonary metastatic OS vs. primary OS. After that, 3 DEmiRNAs (miR-223-5p, miR-378b, and miR-323b-3p), 15 DElncRNAs, and 100 DEmRNAs were obtained (Fig. 5a–c). Based on these above 15 DElncRNAs and 100 DEmRNAs, the target lncRNAs and mRNAs of these three DEmiRNAs (miR-223-5p, miR-378b, and miR-323b-3p) were searched. Then, a total of eight target pairs, including miR-223-5p and its target CLSTN2, AC009951.1 (ENSG00000279166), ENSG00000232679 (LINC01705), and AC090673.1 (ENSG00000197301); miR-378b and its target ALX4, IGSF3, SULF1; miR-323b-3p and target TGFBR3 were obtained. All these miRNAs, lncRNAs, and mRNAs in these eight target pairs were differentially expressed between primary OS and normal control as well as between pulmonary metastasis and primary OS, which involve with both oncogenesis and pulmonary metastasis of OS. Interestingly, their expression pattern was just the opposite in pulmonary metastatic OS vs. primary OS compared with primary OS vs. control (Fig. 5d–f). Pathway analysis of target genes regulated by miR-223-5p, miR-323b-3p, and miR-378b are displayed in Fig. 5g–i.

### qRT-PCR and cross-validation

**Fig. 6:**
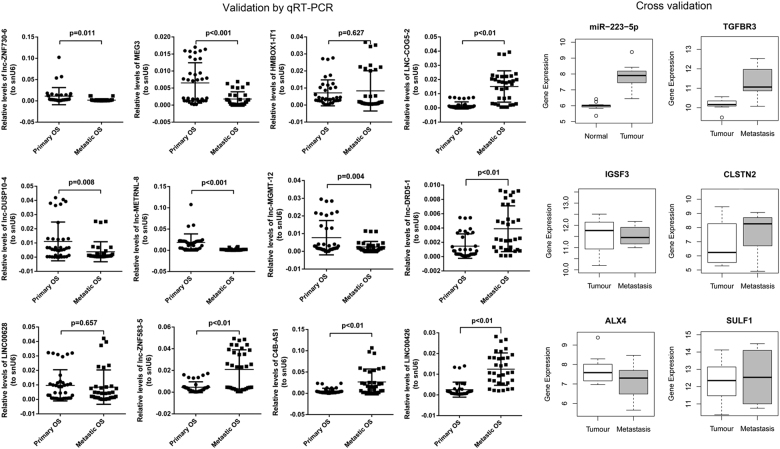
Expression pattern and potential functions of key miRNAs, lncRNAs, and mRNAs in OS progression. **a**–**c** Venn diagram analysis of DEmiRNAs, DElncRNAs, and DEmRNAs in primary OS vs. control and pulmonary metastatic OS vs. primary OS. **d**–**f** Definite expression of key miRNAs, lncRNAs, and mRNAs and their dynamic expression trends. The definite expression was shown based on log_10_(FPKM) or log_10_(normalized read counts) value. **g**–**i** Circos plots of the pathways predicted to be targeted by miR-223-5p (**g**), miR-323b-3p (**h**), and miR-378b (**i**)

Twelve lncRNAs randomly selected from top 15 DElncRNAs between pulmonary metastatic and primary OS were validated by qRT-PCR. Relative expression of DElncRNAs was determined by quantitative RT-PCR of 36 primary OS samples and 33 pulmonary metastatic OS samples (Fig. 6). Expression of these 12 DElncRNAs was consistent with that in RNA-seq results in this study.

miRNA was validated using GSE65071 from GEO database (20 plasma from OS cases and 15 controls plasma). DEmiRNA (miR-223-5p) validated by GSE65071 were randomly selected from three shared DEmiRNAs (miR-223-5p, miR-378b, and miR-323b-3p) in pulmonary metastatic OS vs. primary OS and primary OS vs. normal controls (Fig. 6). Expression of miR-223-5p was consistent with that in RNA-seq results in this study.

Genes were validated by GSE14359 from GEO database (10 conventional OS tissues and eight OS lung metastasis tissues). Five genes validated by GSE14359 were randomly selected from targets of miR-223-5p, miR-378b, and miR-323b-3p which were DEmRNAs in pulmonary metastatic OS vs. primary OS and primary OS vs. normal controls (Fig. 6). Expression of these five genes was consistent with that in RNA-seq results in this study.

### The central role of TGF-β superfamily co-receptor TGFBR3 in OS tumorigenesis and pulmonary metastatic OS

**Fig. 7:**
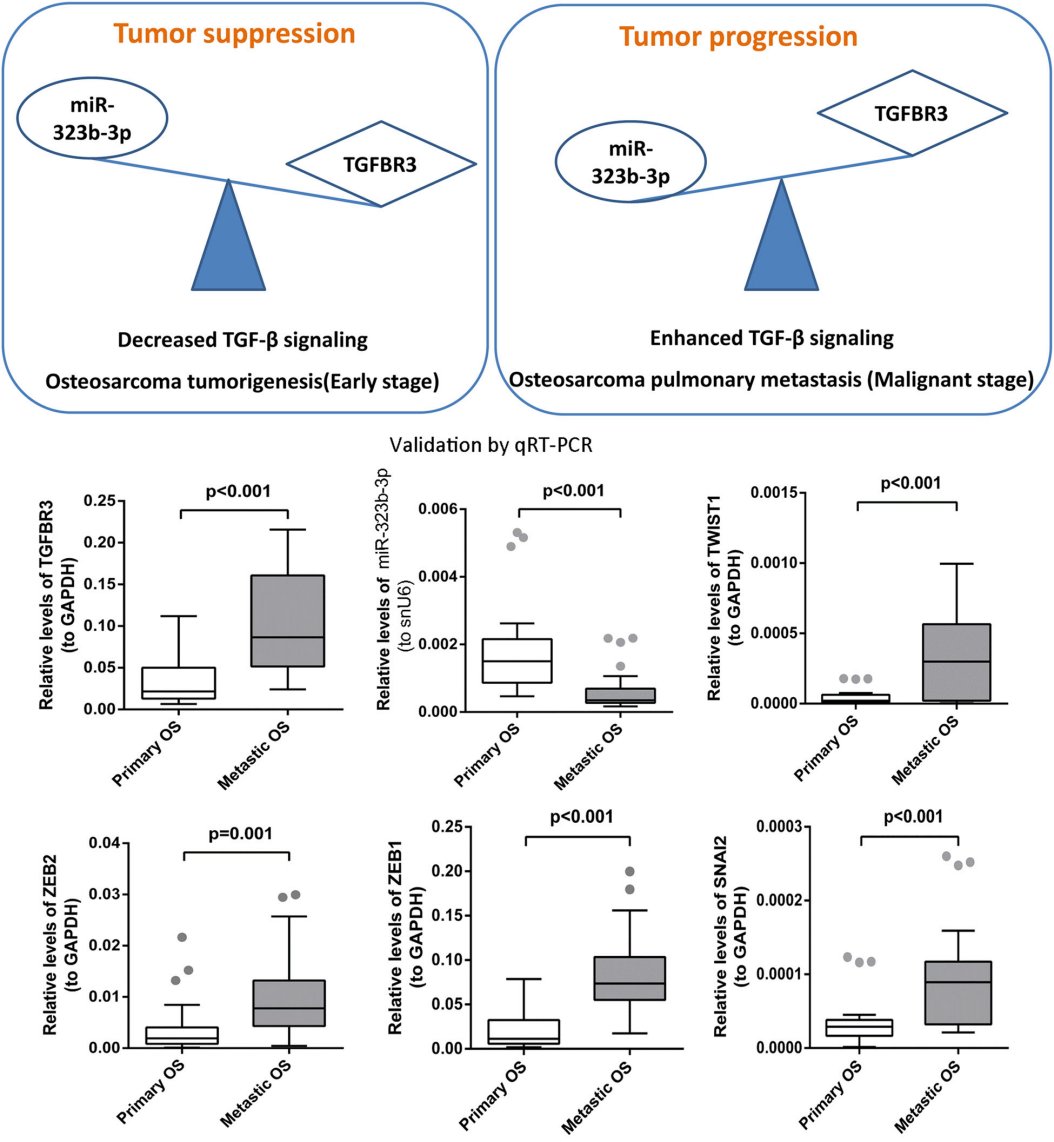
The central role of TGFBR3 in the OS development. TGFBR3 acts as a tumor suppressor in OS tumorigenesis, whereas TGFBR3 acts as a tumor promoter in OS pulmonary metastasis. Expressions of TGFBR3, miR-323b-3p, and four EMT-promoting transcription factors (ZEB1, ZEB2, TWIST1, and SNAI2) have been validated by qRT-PCR. Relative expression was determined by quantitative RT-PCR of 30 primary OS samples and 27 pulmonary metastatic OS samples

Transforming growth factor-β (TGF-β) plays critical roles in the vicious cycle between OS cells and the bone tumor microenvironment, thus contributing to tumor development and lung metastases dissemination^65^. Our RNA-seq results showed that TGFBR3, a TGF-β superfamily co-receptor, was decreased in the process of OS tumorigenesis and increased in the process of OS pulmonary metastasis. In addition, TGFBR3 was a target of miR-323b-3p, and miR-323b-3p was increased in OS development while decreased in pulmonary metastatic OS based on our RNA-seq results. Moreover, both upregulated TGFBR3 and downregulated miR-323b-3p were observed in pulmonary metastatic OS compared to primary OS in our qRT-PCR results (Fig. 7). We established the central role of TGFBR3 in the OS development, which was shown in Fig. 7. At the early stage, miR-323b-3p inhibits the expression of TGFBR3, which acts as tumor suppressors in OS tumorigenesis by decreasing the TGF-β signaling. However, at the malignant stage, miR-323b-3p promotes the expression of TGFBR3, which acts as tumor promoters in pulmonary metastatic OS by enhancing the TGF-β signaling (Fig. 7). Previous study revealed that TGF-βs may act as a tumor suppressor by inhibiting the proliferation of epithelial cells and act as tumor promoters during the late stages of carcinogenesis by inducing epithelial–mesenchymal transition (EMT), to stimulate angiogenesis, and to favor immune evasion^65^. Hence, expression of EMT-promoting transcription factors, including ZEB1, ZEB2, TWIST1, and SNAI2^66^, was validated by qRT-PCR. All these four EMT-promoting transcription factors were elevated in pulmonary metastatic OS compared to primary OS based on qRT-PCR results (Fig. 7).

## Discussion

OS is the most common pediatric malignant bone tumor with early pulmonary metastasis formation as a frequent occurrence. Once metastasized to the lung, OS generally causes a rapid and fatal outcome. To improve this situation, increasing attention has been given to identify the exact regulatory mechanism of OS development and malignancy. Recent years, ncRNAs have been found to be associated with wide range of biological regulatory functions^67^. The present study utilized next-generation sequencing to provide a quantitative and comprehensive analysis of the coding and non-coding transcriptome in primary OS and pulmonary metastasis. These analyses revealed significant differences in the patterns of miRNA, lncRNA, and mRNA expression in primary OS and pulmonary metastasis, as well as the dynamic changes of DEmiRNA, DElncRNA, and DEmRNA. Here we showed for the first time that the expression patterns of lncRNAs and mRNAs are more suitable to discriminate the controls, primary OS, and pulmonary metastatic OS samples.

In general, our data suggest that distinct populations of miRNAs, lncRNAs, and mRNAs are involved in the pathogenesis of primary OS and OS pulmonary metastasis. Based on the RNA sequence data, we identified 65 miRNAs, 233 lncRNAs, and 1405 mRNAs were differentially expressed in primary OS compared with the normal controls. We found that most of the DEmiRNAs may be associated with OS tumorigenesis. However, most of the DElncRNAs were with unknown function, which is mainly due to the few researches for them. Based on tumorigenesis-related ceRNAs, Apoptosis and MAPK signaling pathway were two significantly enriched pathways which were well-established cancer-related pathways^47,48^. PPP3CC, protein phosphatase 3 catalytic subunit gamma was a shared gene in these two pathways, decreased PPP3CC has been found in prostate cancer and gliomas^68,69^. We firstly found downregulation of PPP3CC in primary OS compared to normal controls in this study. Moreover, PPP3CC was a neighboring gene of a downregulated lncRNA in primary OS compared to normal controls, AC037459.2 (ENSG00000251034). PPP3CC-AC037459.2 interaction was speculated to involve with the processes of OS and other cancers by regulating Apoptosis and MAPK signaling pathway. The precise role of PPP3CC-AC037459.2 interaction in cancers needs further research. Besides, dysregulated mRNAs in the OS tumorigenesis-related ceRNA network were mainly involved in another two pathways, Phagosome and ECM-receptor interaction that highlighted their importance in OS. Recent studies identified that miRNA interactions with lncRNA and mRNA might play important roles in OS formation, pulmonary metastasis and prognosis, such as miR-30a^70^, miR-136^71^, miR-206^10^, miR-181b^32^, miR-29b-3p^72^, miR-29a^73^, miR-133a^74^, miR-224^30^, and miR-223^24^. In our ceRNA network, we also found these key miRNAs have most target lncRNAs or mRNAs. Therefore, our results suggested that these key miRNAs may play an important role in the progression and development of OS and the cancer genes related pathways.

Subsequently, we detected 48 miRNAs, 50 lncRNAs, and 307 mRNAs that had different expression patterns in pulmonary metastatic OS compared with primary OS. DEmRNAs in OS pulmonary metastasis-related ceRNA network were significantly enriched in a well-known cancer-related pathway, p53 signaling pathway^64^. SESN2, sestrin 2 was enriched in this pathway which was reported to involve with various cancers such as bladder, breast, and lung cancers^75–77^. In this study, SESN2 was firstly found to be upregulated in pulmonary metastatic OS compared to primary OS. These findings suggested that SESN2 might be a potential tumor suppressor of OS by regulating p53 signaling pathway.

Among which, the top ten miRNAs were considered as the most important ones participating in OS pulmonary metastasis. Previous studies also indicated that their dysregulation may contribute to the progression or OS metastasis, such as miR-20b^58^, miR-182^60^, miR-134^78^, and miR-183^79^. Our results first suggested that miR-612, miR-1197, miR-193b-3p, miR-1262, miR-144-3p, and miR-1269a may also play roles in OS metastasis.

Moreover, a total of three DEmiRNAs were inferred as the most promising candidate genes affecting OS development, which were further described as follows. Namløs et al. found that miR-223 was identified with an intermediate expression level in OS clinical samples compared to osteoblasts and bone^80^. MiR-223 may have a tumor suppressor function in OS through the PI3K/Akt/mTOR pathway and could be used in anticancer therapies in OS^81^. miR-223/Ect2/p21 signaling is also an important pathway that regulates the OS cell cycle progression and proliferaion^82^. Combination of miR-223 downregulation and Ect2 upregulation may be a possible marker of poor prognosis in OS malignancy^24,83^. In our present study, we found that miR-223-5p was upregulated in primary OS, whereas downregulated in OS pulmonary metastasis. The results suggested that miR-223-5p may act as important roles in OS development. Grilli et al. observed the modulation of miR-378 using an OS differentiative model^15^. Novello et al. also found that miR-378 was significantly downregulated in OS vs. control, high-grade OS vs. low-grade OS, and metastatic OS vs. non‑metastatic OS patients by RT-PCR^84^. Our data showed that miR-378b was downregulated in primary OS, whereas upregulated in OS pulmonary metastasis. These observations also suggested that miR-378b may be essential in OS progression. Although the relationship of miR-323b-3p and OS was not reported, our study showed a significant dysregulation of miR-323b-3p during the OS development and malignancy, which implied its critical roles in OS.

TGF-β signaling pathway is critical in OS development and in their metastatic progression^65^. TGF-βs act as both tumor suppressors and tumor promoters, depending on the cancer type and tumor development timing^85^. Previous study revealed that TGF-βs may act as a tumor suppressor by inhibiting the proliferation of epithelial cells and act as tumor promoters during the late stages of carcinogenesis by inducing EMT, to stimulate angiogenesis, and to favor immune evasion^65^. Moreover, the increase of TGF-βs is also associated with the presence of metastases in lung^86^ and is correlated with high-grade OS^87^. TGFBR3 is a co-receptor for the TGF-β superfamily, which can present ligand to the TGF-β signaling receptors. TGFBR3 is a tumor suppressor in many tissue types^88^. Our RNA-seq results found that TGFBR3 is decreased in OS development and increased in pulmonary metastatic OS which was consistent with that in both the cross-validation and qRT-PCR validation. Moreover, upregulated expression of several EMT-promoting transcription factors including ZEB1, ZEB2, TWIST1, and SNAI2 was found in pulmonary metastasis compared to primary OS based on our qRT-PCR validation results. We suggested that TGFBR3 acts as a tumor suppressor during the early stage of OS development and becomes a tumor promoter during the late stages of metastases via activation of the EMT program. Therefore, blocking TGF-β signaling may represent a promising therapeutic approach to treat OS patients. Additionally, TGFBR3 was a target of miR-323b-3p and miR-323b-3p was found to be upregulated in OS development while downregulated in pulmonary metastatic OS based on RNA-seq, cross-validation, and qRT-PCR validation results in this study. We speculated that TGF-β signaling pathway was regulated by miR-323b-3p in oncogenesis and metastasis of OS.

Our study has some limitations. The number of samples analyzed here was relatively small, and the samples were obtained from a heterogeneous cohort of patients and donors. It may introduce some bias. Moreover, little is known about the alteration and functional significance of lncRNAs and additional studies are needed to explore the functional roles of lncRNAs in OS. Taken together, our study revealed distinct relative abundance and expression pattern of miRNAs, lncRNAs, and mRNAs in human OS, highlighting the different biological roles of the individual RNA classes during OS progression. Our results provide valuable information for ncRNAs studies in the future.

## Electronic supplementary material

Supplemental Table S1

Supplemental Table S2

Supplemental Figure 1

Supplemental Figure 2

Supplemental Figure 3

Supplemental Figure 4

Supplemental Figure 5

Supplementary figure legends
