# Supplementary material for: Deep RNA sequencing reveals the dynamic regulation of miRNA, lncRNAs, and mRNAs in osteosarcoma tumorigenesis and pulmonary metastasis
Source: Cell Death Dis. 2018 Jul 10;9(7):772. doi: 10.1038/s41419-018-0813-5 (PMC6039476; doi:10.1038/s41419-018-0813-5)
Supplement: Supplementary file 1 — Supplemental Table S1 [file 41419_2018_813_MOESM1_ESM.doc]

Supplemental Table S1 Deep RNA sequencing of subjects

| Sample ID | | lncRNA+mRNA sequencing | | | miRNA sequencing | | |
| --- | --- | --- | --- | --- | --- | --- | --- |
|  |  | Clean reads | Q30(%) | Mapped Ratio | Clean reads | Q30% | Mapped Ratio |
| primary  OS | T1 | 83129306 | 91.83;86.78 | 77.37% | 10074451 | 96.47 | 47.25% |
|  | T2 | 81499206 | 92.66;91.88 | 88.35% | 18253213 | 98.13 | 79.10% |
|  | T3 | 80542254 | 92.41;91.85 | 90.20% | 13137397 | 96.95 | 98.17% |
| OS pulmonary metastasis | M1 | 104337490 | 92.32;88.09 | 81.63% | 11784463 | 96.62 | 53.12% |
|  | M2 | 93048830 | 92.47;88.66 | 78.36% | 8115544 | 96.8 | 58.10% |
|  | M3 | 80933392 | 92.01;91.76 | 90.63% | 15006164 | 98.13 | 38.15% |
| Normal control | N1 | 96860392 | 92.73;89.29 | 75.50% | 13418146 | 97.25 | 70.21% |
|  | N2 | 87156636 | 93.02;88.09 | 85.64% | 10852394 | 97.44 | 47.74% |
|  | N9 | 80506954 | 92.56;92.39 | 89.67% | 12118799 | 97.34 | 95.24% |
