# Supplementary material for: Deep RNA sequencing reveals the dynamic regulation of miRNA, lncRNAs, and mRNAs in osteosarcoma tumorigenesis and pulmonary metastasis
Source: Cell Death Dis. 2018 Jul 10;9(7):772. doi: 10.1038/s41419-018-0813-5 (PMC6039476; doi:10.1038/s41419-018-0813-5)
Supplement: Supplementary file 2 — Supplemental Table S2 [file 41419_2018_813_MOESM2_ESM.doc]

**Supplemental Table S1 Significantly enriched KEGG pathway of mRNAs in OS tumorigenesis-related ceRNA network and OS pulmonary metastasis-related ceRNA network**

| **Primary OS vs. normal controls** | | | |
| --- | --- | --- | --- |
| **KEGG ID** | **Pathways** | **FDR** | **Enriched Genes** |
| Kegg:04145 | Phagosome | 1.20E-08 | ITGAM, CYBA, TUBA1B, MRC2, TUBA1C |
| Kegg:04974 | Protein digestion and absorption | 1.22E-05 | COL1A1, COL6A3, KCNE3 |
| Kegg:04512 | ECM-receptor interaction | 1.87E-05 | COL1A1, AGRN, COL6A3 |
| Kegg:00290 | Valine, leucine and isoleucine biosynthesis | 2.34E-05 | BCAT1 |
| Kegg:05010 | Alzheimer's disease | 0.000112026 | NDUFA5, ATP2A1, PPP3CC, NDUFS7 |
| Kegg:00520 | Amino sugar and nucleotide sugar metabolism | 0.000155126 | UAP1L1, PGM1 |
| Kegg:00790 | Folate biosynthesis | 0.000157775 | ALPL |
| Kegg:05152 | Tuberculosis | 0.000163556 | ITGAX, ITGAM, PPP3CC, MRC2 |
| Kegg:04660 | T cell receptor signaling pathway | 0.000199108 | PPP3CC, PTPRC, LAT |
| Kegg:04670 | Leukocyte transendothelial migration | 0.000303951 | ITGAM, CYBA, MYL12A |
| Kegg:05130 | Pathogenic Escherichia coli infection | 0.000332097 | TUBA1B, TUBA1C |
| Kegg:00533 | Glycosaminoglycan biosynthesis - keratan sulfate | 0.000399644 | ST3GAL3 |
| Kegg:04510 | Focal adhesion | 0.000459345 | FLNC, COL1A1, MYL12A, COL6A3 |
| Kegg:04514 | Cell adhesion molecules (CAMs) | 0.000699092 | ITGAM, PTPRC, VCAN |
| Kegg:04380 | Osteoclast differentiation | 0.000707255 | PPP3CC, CYBA, SPI1 |
| Kegg:00770 | Pantothenate and CoA biosynthesis | 0.000851912 | BCAT1 |
| Kegg:05140 | Leishmaniasis | 0.00128259 | ITGAM, CYBA |
| Kegg:05412 | Arrhythmogenic right ventricular cardiomyopathy (ARVC) | 0.002764 | DSP, SGCA |
| Kegg:04010 | MAPK signaling pathway | 0.00541341 | MEF2C, PPP3CC, FLNC, NR4A1 |
| Kegg:04210 | Apoptosis | 0.00818661 | PPP3CC, CFLAR |
| **Pulmonary metastatic OS vs. Primary OS** | | | |
| **KEGG ID** | **Pathways** | **FDR** | **Enriched Genes** |
| Kegg:00601 | Glycosphingolipid biosynthesis - lacto and neolacto series | 1.08E-06 | ST8SIA1, B3GALT1 |
| Kegg:04971 | Gastric acid secretion | 3.25E-05 | KCNJ15, ADCY1, PLCB4 |
| Kegg:05143 | African trypanosomiasis | 3.95E-05 | APOL1, PLCB4 |
| Kegg:04640 | Hematopoietic cell lineage | 0.00017109 | ITGA3, CD1D, MME |
| Kegg:04970 | Salivary secretion | 0.000185197 | LYZ, ADCY1, PLCB4 |
| Kegg:00740 | Riboflavin metabolism | 0.000223865 | ENPP1 |
| Kegg:04540 | Gap junction | 0.000236996 | ADCY1, PLCB4, PDGFA |
| Kegg:00230 | Purine metabolism | 0.00043893 | AK5, ADCY1, PDE3A, ENPP1 |
| Kegg:04916 | Melanogenesis | 0.000550314 | ADCY1, PLCB4, FZD2 |
| Kegg:05142 | Chagas disease (American trypanosomiasis) | 0.000694528 | ADCY1, PLCB4, C1QC |
| Kegg:05146 | Amoebiasis | 0.000694528 | ADCY1, PLCB4, CD1D |
| Kegg:00603 | Glycosphingolipid biosynthesis - globo series | 0.00113165 | ST8SIA1 |
| Kegg:00533 | Glycosaminoglycan biosynthesis - keratan sulfate | 0.00167089 | B3GNT7 |
| Kegg:00604 | Glycosphingolipid biosynthesis - ganglio series | 0.00167089 | ST8SIA1 |
| Kegg:04510 | Focal adhesion | 0.00264302 | ITGA3, TNN, PDGFA, VAV3 |
| Kegg:04724 | Glutamatergic synapse | 0.00319679 | ADCY1, SLC38A1, PLCB4 |
| Kegg:00770 | Pantothenate and CoA biosynthesis | 0.00323178 | ENPP1 |
| Kegg:04614 | Renin-angiotensin system | 0.00323178 | MME |
| Kegg:04610 | Complement and coagulation cascades | 0.00463709 | C1QC, PLAU |
| Kegg:04115 | p53 signaling pathway | 0.00552229 | GADD45A, SESN2 |
