# Supplementary figures and images for: Deep RNA sequencing reveals the dynamic regulation of miRNA, lncRNAs, and mRNAs in osteosarcoma tumorigenesis and pulmonary metastasis
Source: Cell Death Dis. 2018 Jul 10;9(7):772. doi: 10.1038/s41419-018-0813-5 (PMC6039476; doi:10.1038/s41419-018-0813-5)

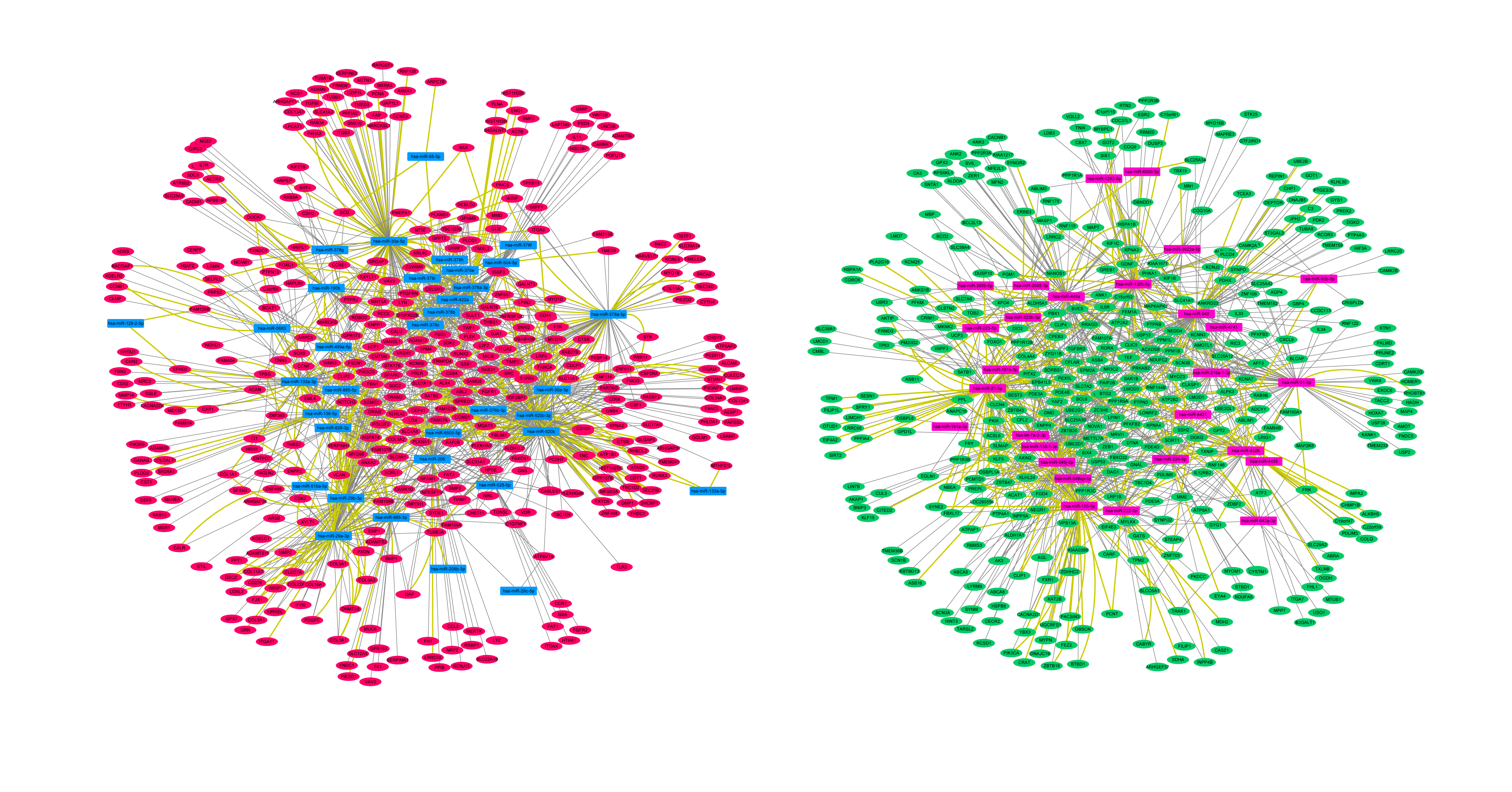

Supplement: Supplementary file 3 — Supplemental Figure 1 [file 41419_2018_813_MOESM3_ESM.tif]

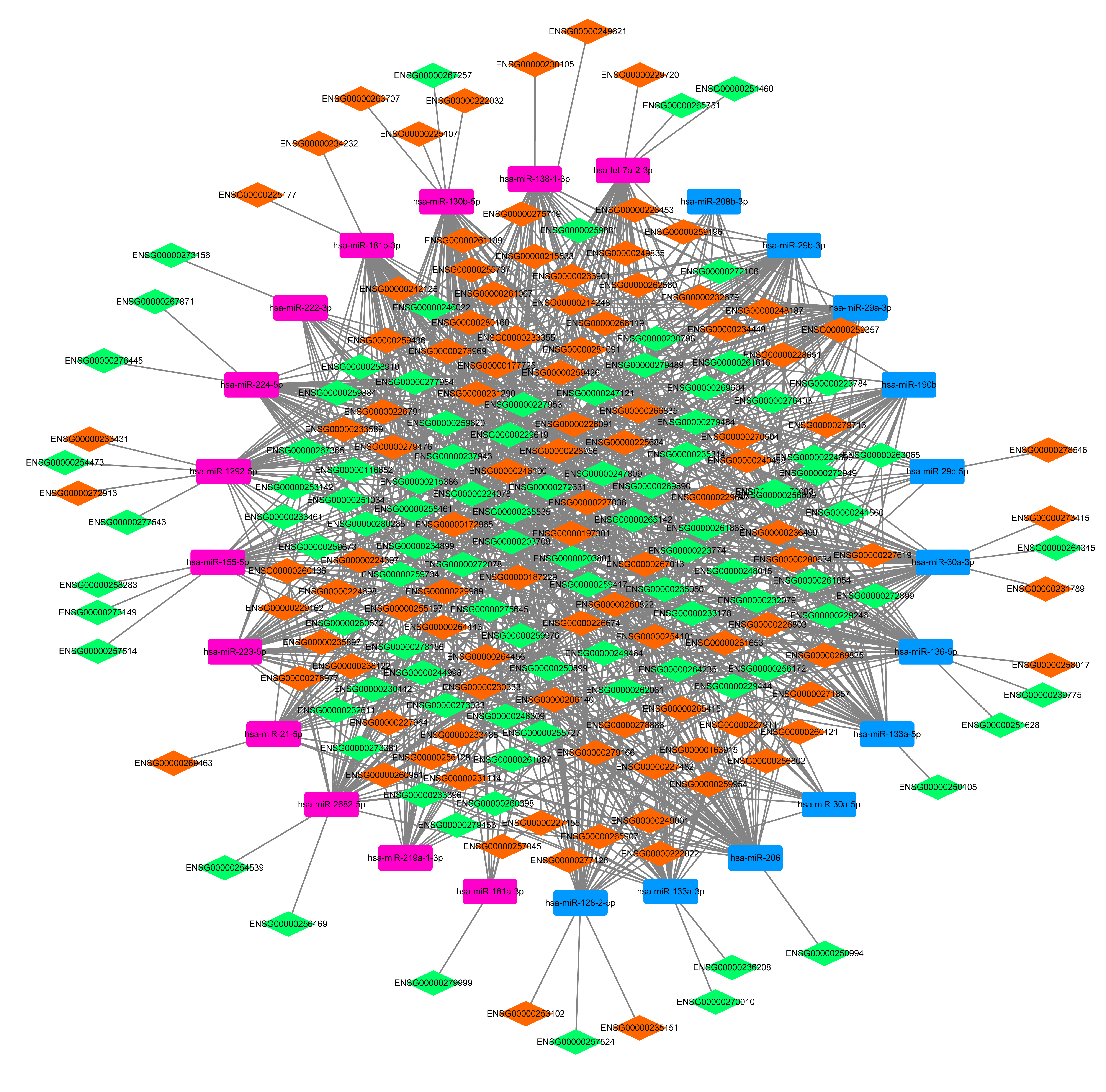

Supplement: Supplementary file 4 — Supplemental Figure 2 [file 41419_2018_813_MOESM4_ESM.tif]

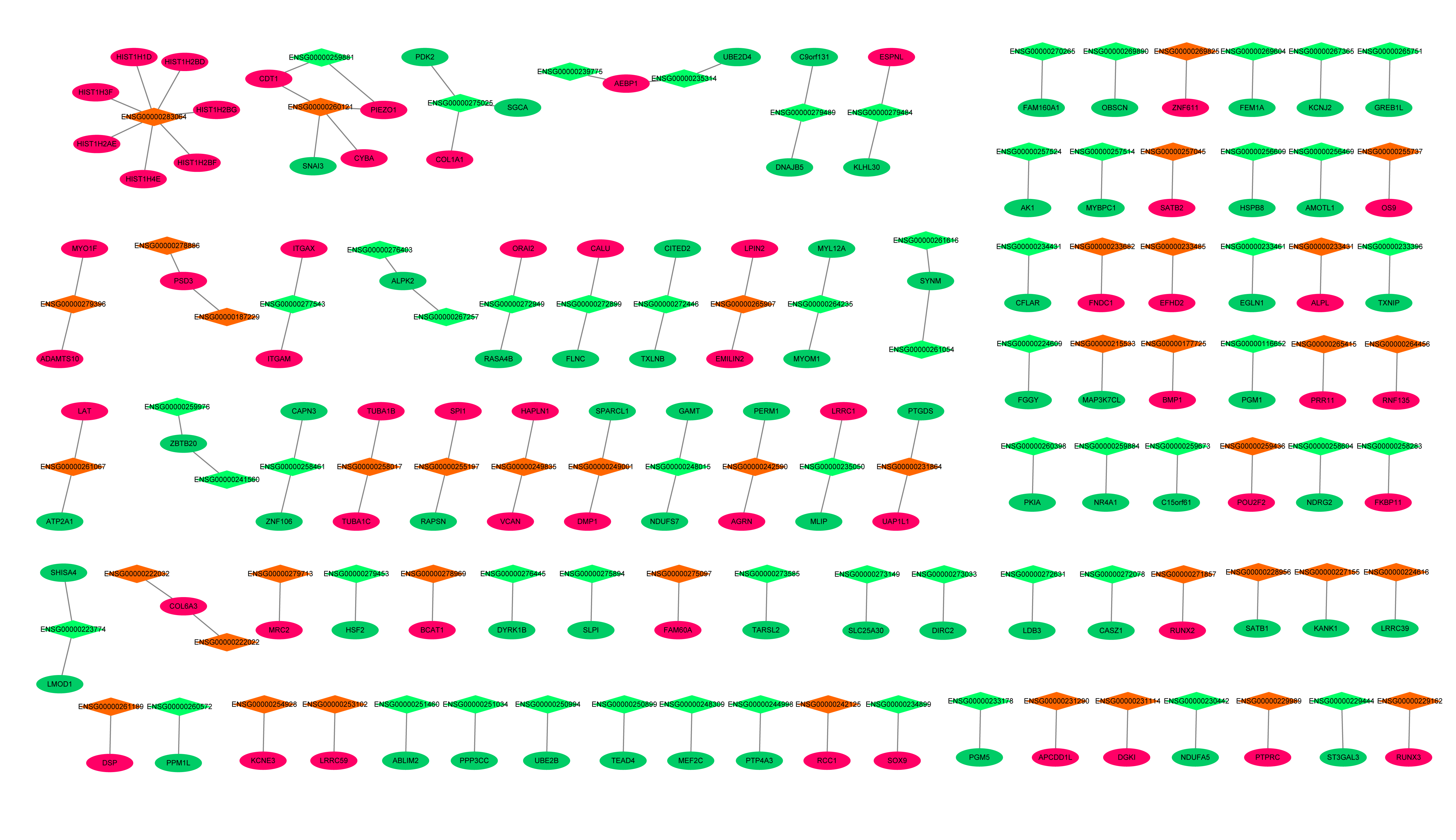

Supplement: Supplementary file 5 — Supplemental Figure 3 [file 41419_2018_813_MOESM5_ESM.tif]

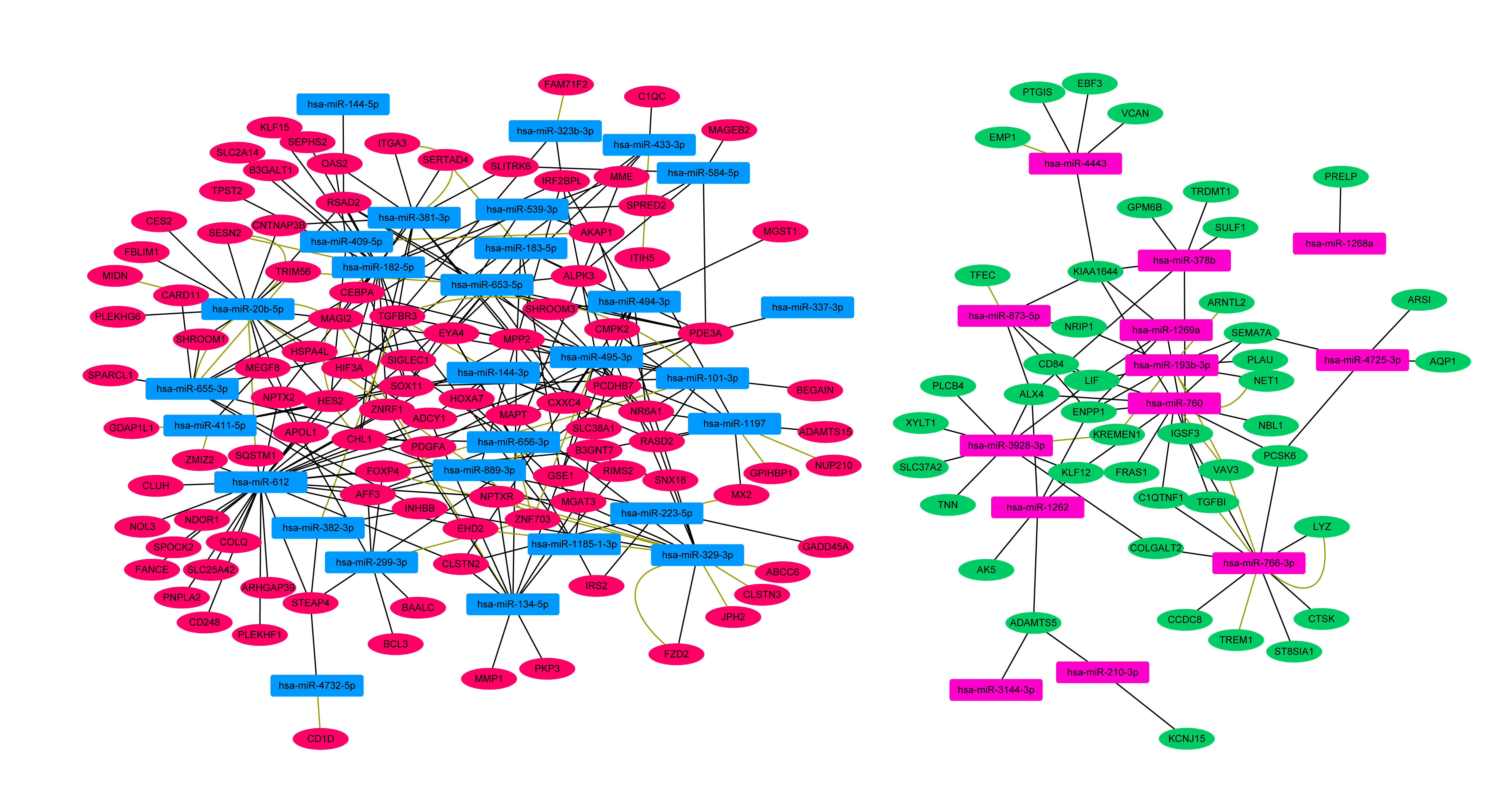

Supplement: Supplementary file 6 — Supplemental Figure 4 [file 41419_2018_813_MOESM6_ESM.tif]

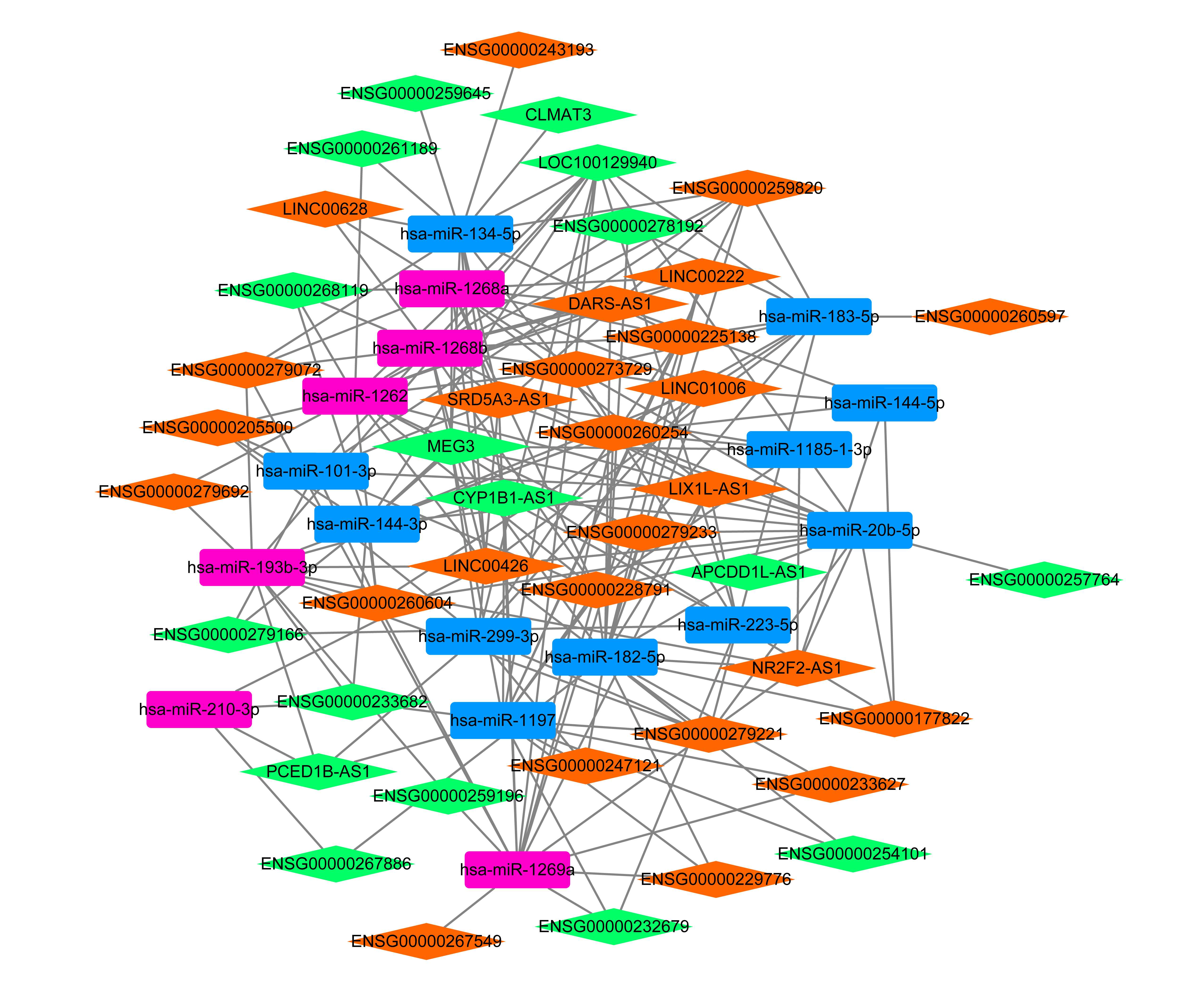

Supplement: Supplementary file 7 — Supplemental Figure 5 [file 41419_2018_813_MOESM7_ESM.tif]
