# Supplementary material for: Deep RNA sequencing reveals the dynamic regulation of miRNA, lncRNAs, and mRNAs in osteosarcoma tumorigenesis and pulmonary metastasis
Source: Cell Death Dis. 2018 Jul 10;9(7):772. doi: 10.1038/s41419-018-0813-5 (PMC6039476; doi:10.1038/s41419-018-0813-5)
Supplement: Supplementary file 8 — Supplementary figure legends [file 41419_2018_813_MOESM8_ESM.doc]

**Supplemental Figure 1：OS tumorigenesis-related miRNA-mRNA target pairs**

Blue and Rose red rectangles represented down- and up-regulated miRNAs, respectively. Green and pink ellipses represented down- and up-regulated mRNAs, respectively.

**Supplemental Figure 2:：OS tumorigenesis-related miRNA-lncRNA target pairs**

Blue and Rose red rectangles represented down- and up-regulated miRNAs, respectively. Green and orange rhombuses represented down- and up-regulated lncRNAs, respectively.

**Supplemental Figure 3：OS tumorigenesis-related lncRNA-mRNA target pairs**

Green and pink ellipses represented down- and up-regulated mRNAs, respectively. Green and orange rhombuses represented down- and up-regulated lncRNAs, respectively.

**Supplemental Figure 4：OS pulmonary metastasis-related miRNA-mRNA target pairs**

Blue and Rose red rectangles represented down- and up-regulated miRNAs, respectively. Green and pink ellipses represented down- and up-regulated mRNAs, respectively.

**Supplemental Figure 5：OS pulmonary metastasis-related miRNA-lncRNA target pairs**

Blue and Rose red rectangles represented down- and up-regulated miRNAs, respectively. Green and orange rhombuses represented down- and up-regulated lncRNAs, respectively.
